# A cellulose binding domain protein restores female fertility when expressed in transgenic Bintje potato

**DOI:** 10.1186/s13104-016-1978-6

**Published:** 2016-03-18

**Authors:** Richard W. Jones, Frances G. Perez

**Affiliations:** Genetic Improvement of Fruits and Vegetables Laboratory, USDA-ARS, 10300 Baltimore Ave., Beltsville, MD 20705 USA

**Keywords:** Cellulose binding domain, Bintje, Potato fertility

## Abstract

**Background:**

Expression of a gene encoding the family 1 cellulose binding domain protein CBD1, identified in the cellulosic cell wall of the potato late blight pathogen *Phytophthora infestans*, was tested in transgenic potato to determine if it had an influence on plant cell walls and resistance to late blight.

**Results:**

Multiple regenerants of potato (cv. Bintje) were developed and selected for high expression of CBD 1 transcripts. Tests with detached leaflets showed no evidence of increased or decreased resistance to *P. infestans*, in comparison with the blight susceptible Bintje controls, however, changes in plant morphology were evident in CBD 1 transgenics. Plant height increases were evident, and most importantly, the ability to produce seed berries from a previously sterile cultivar. Immunolocalization of CBD 1 in seed berries revealed the presence throughout the tissue. While Bintje control plants are male and female sterile, CBD 1 transgenics were female fertile. Crosses made using pollen from the late blight resistant Sarpo Mira and transgenic CBD1 Bintje as the female parent demonstrated the ability to introgress *P. infestans* targeted resistance genes, as well as genes responsible for color and tuber shape, into Bintje germplasm.

**Conclusions:**

A family 1 cellulose-binding domain (CBD 1) encoding gene from the potato late blight pathogen *P. infestans* was used to develop transgenic Bintje potato plants. Transgenic plants became female fertile, allowing for a previously sterile cultivar to be used in breeding improvement. Selection for the absence of the CBD transgene in progeny should allow for immediate use of a genetically enhanced material. Potential for use in other Solanaceous crops is proposed.

## Background

Cellulose binding domains (CBDs) represent a subset of carbohydrate binding modules (CBMs). CBDs are found associated with the majority of saprophyte-encoded cellulolytic enzymes, whereas they are generally not found associated with plant pathogen-encoded or with plant-encoded cellulolytic enzymes [1, 2]. The principle function of the CBD is to mediate adherence to the carbohydrate substrate [3, 4], while an additional function can be found in the ability to of some CBDs to directly dissociate cellulose microfibrils [5]. Within the CBD families, there are a few examples of CBDs that are present as independent proteins, often associated with the cell wall. An example is CBD 1 from the potato late blight disease inducing phytopathogen *Phytophthora infestans* [6]. The *P. infestans* CBD 1 protein is found tightly associated with the cellulose-containing hyphal cell wall, and may play a role in assembly and/or integrity of the cell wall. This led us to the idea that expression of this protein in potato plants may alter the host: pathogen interactions through binding to the host cellulose molecules, proving a potential shield from pathogen endoglucanases, or conversely, binding to *Phytophthora* hyphae, providing a shield from host endoglucanases. We report the unexpected outcome of *P. infestans* CBD 1 expression in transgenic Bintje potato plants, restoration of female fertility. We demonstrate the usefulness of the fertility through crosses resulting in late blight resistant plants with expanded tuber colors.

## Methods

### Plant transformation, selection and characterization

Transgenic potato (cv. Bintje) was developed using 4 week old tissue culture grown plantlets. Leaves were excised, cut laterally and immersed for 15 min in an acetosyringone-induced (100 μM) culture of *Agrobacterium tumefaciens* LBA4404 containing the *CBD 1* gene (EU179903) in the binary vector pBI121. Subsequent procedures were essentially as reported previously [7]. Regenerated plantlets were maintained in a growth chamber on MS media. DNA samples from putative transformants were screened by PCR using 35S specific primers (35S-F GATAATCATCGCAAGACCGGC and 35S-R GACGTAAGGGATGACGCACAATCCC) followed by sequencing of the PCR product. Plantlets of uniform height were transferred to soil and maintained in a greenhouse. Height measurements were recorded weekly beginning at 5 weeks, when all plants were established.

Integration sites were determined for some plants using the APAgene GOLD Genome Walking Kit (BioS&T, Montreal, Canada) according to manufacturer’s protocols. Forward primer sequences, used in sequential PCR reactions, were TTACCCAACTTAATCGCCTTGCAGC, TGGCCGTCGTTTTACAACGTCGTGAC and CGTCGTGACTGGGAAAACCCTGG, corresponding to regions from the 3′ end of CBD1 and the T-DNA border of pBI121. Final reactions were separated on an agarose gel, purified and cloned (TOPO-TA, Invitrogen) followed by sequencing.

Immunolocalization of CBD 1 was performed on developing seed berry cross sections. Samples were fixed and rehydrated as previously described, followed by antibody localization as previously described, using peptide-specific CBD 1 antibodies [6]. Secondary goat anti-rabbit antibody conjugated to alkaline phosphatase (Sigma) was used at a 1:1000 dilution, followed by two rinses with Tris buffer (pH 9.5), followed by visualization with Western blot AP substrate (Promega). Control samples were treated with rabbit pre-immune serum, followed by the secondary goat anti-rabbit AP antibody and treatment with AP substrate.

### Late blight resistance screening

Sporangia were harvested from 2 week old cultures of *P. infestans* (race US 11) by flooding plates with 5 ml sterile water and decanting the sporangia into a sterile petri dish. Harvested sporangia were refrigerated for 1 h, followed by incubation at room temperature for 30 min to induce zoospore formation. Leaflets from 10 week old greenhouse grown potato plants were detached from the center of the plants and placed onto moistened paper towels in incubation trays. Fifty microliter aliquots of the sporangia/zoospore mixture were applied at individual sites on the abaxial side of the leaves. Incubation trays were sealed with plastic wrap and placed in an incubator (18 C). Inoculated leaves were kept in the dark for 24 h, followed by 14 h light/10 dark lighting cycles. Disease progress was scored relative to control Bintje leaflets.

### Breeding studies

During an initial round of transgenic plant assessment it was noticed that the transgenic lines formed seed berries, unlike the control plants where the flowers abscised after bloom. However, the mature seed berries did not contain seed. Crosses were made using Bintje as the female parent. Pollen from the late blight resistant cultivar Sarpo Mira [8], or pollen from the purple skin, yellow fleshed cultivar Peter Wilcox were applied to the stigmas of Bintje transgenics B-7 and B-48, and a control non-transformed Bintje. Pollen production was extremely limited in both control and transformed Bintje, and pollen was non-viable so Bintje could not be used as a male parent. Seed berries were allowed to mature and the seed was harvested and dried. A subset of seed from each of the successful cross pollinations was planted to assess progeny. Screening for late blight resistance followed the same protocol as outlined earlier in this paper.

## Results

### Characterization of CBD 1 integration and expression

**Fig. 1:**
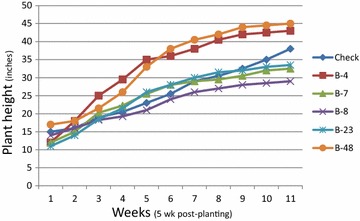
Height of *CBD*1 transgenic and control plant beginning 5 weeks after transfer to soil in the greenhouse. Only certain lines had height increases noticeably exceeding the control plant

**Fig. 2:**
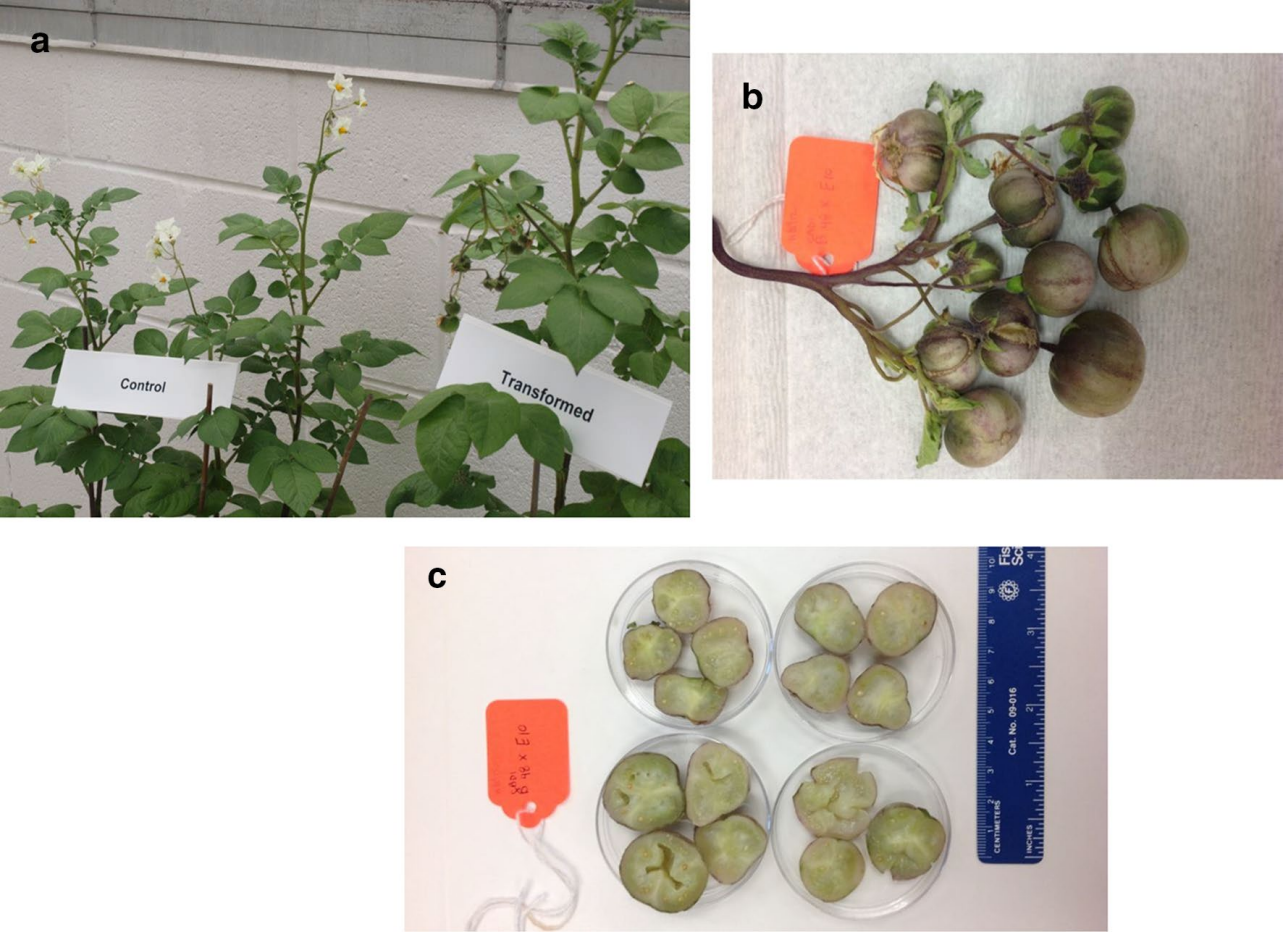
Floral and seed berry formation on Bintje *CBD* 1 transgenic. **a** Earlier flowering seen by the presence of seed berries in transformed plant B7 that is the same age as control plants where they have just initiated flowering **b** abundant seed berries **c** largest seed berries were produced from B-48, producing as many as 200 seeds/berry after outcrossing

**Fig. 3:**
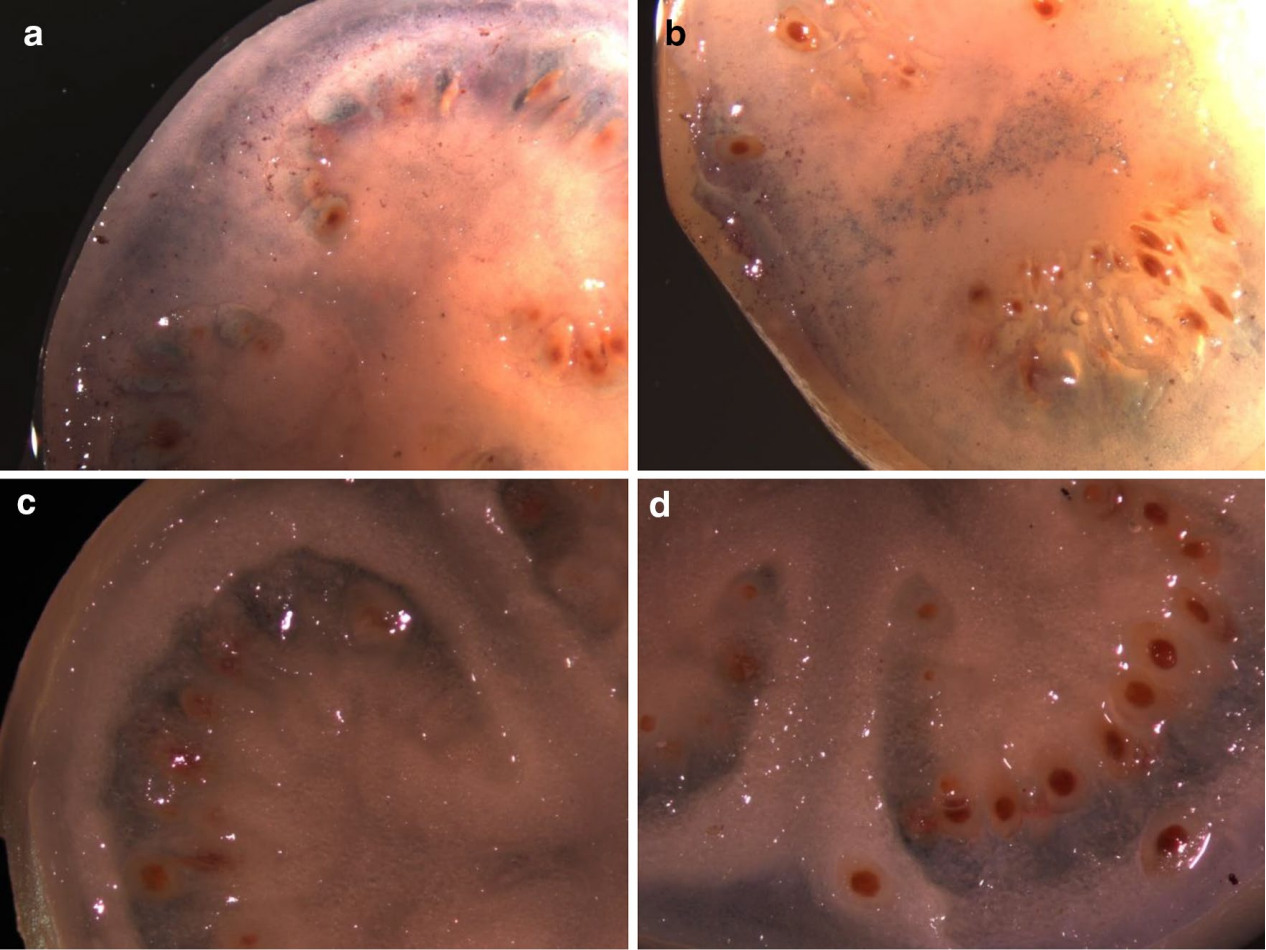
Immunolocalization of CBD 1. *Purple regions* represent precipitation of AP substrate. **a**, **b** CBD 1 protein can be seen accumulating throughout the fleshy region of the ovary and locule. **c**, **d** No purple precipitant is observed with transgenic tissues when using rabbit pre-immune serum followed by goat-anti rabbit AP conjugated secondary antibody, then AP substrate exposure

Height increases were evident in some transgenic lines (Fig. 1). Transgenic Bintje plants produced numerous seed berries, each with viable seeds only when out-crossed with donor pollen (Fig. 2). The presence of seed berries was uniform, while some transgenics, such as B48 had larger seed berries. Immunolocalization revealed abundant levels of CBD 1 protein dispersed throughout the wall and locule tissues of all the developing seed berries (Fig. 3). The CBD 1 protein was found in seed berry samples of all transgenics (developed without pollination) and in transgenic B7 And B48 seed berries after fertilization with donor pollen.

**Fig. 4:**
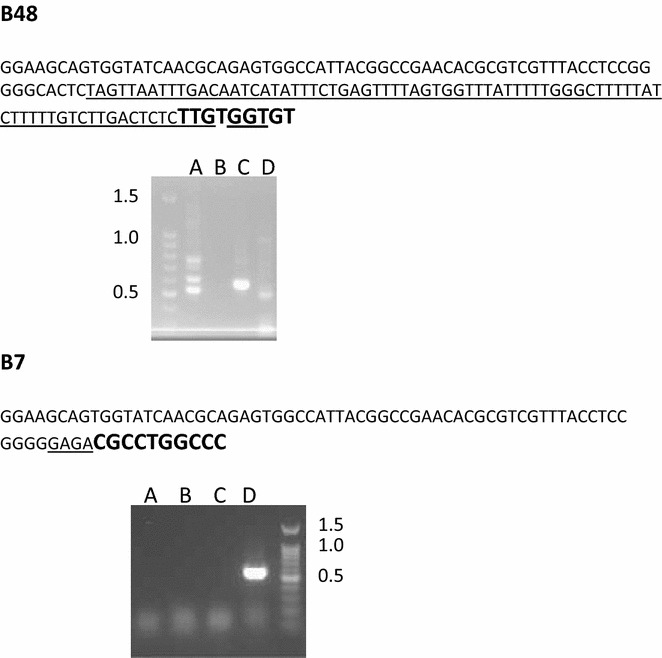
Genome walking identification of integration sites using *CBD* 1 gene and vector specific forward primers and four sets of ApaGene reverse primers labeled *A*, *B*, *C* and *D*. After three rounds of PCR a single band was evident for **B7**, while multiple bands were present in the **B48** samples. Band sizes greater than 500 bp are needed to identify integration sequences. *Large bold* *sequence* represents the pBI121 T-DNA *border region*, *underlined sequences* represent unique potato sequence, remaining sequence is common to APAgene kit

Genome integration sites for the two transgenics used in the crossing studies (B7 and B48) were identified through genome walking. Based on the differing pattern of bands, and the difference in sequence at the integration sites they can be seen as unique transgenic events (Fig. 4). The B7 integration occurred with a more truncated left border region, compared with the B48 transgenic. There was insufficient sequence corresponding to the integration site for B7 to determine the location, however it would likely be a single integration based on the single band generated, and the sequence was different from the B48 integration region. The B48 transgenic generated multiple bands within and between genome walking primer sets. The only potato sequence identified was the region 1071,661–1071,733 of NW_006239126. This region corresponds to the middle of a 1089 bp intron located between two exons for a putative ODORANT 1 protein mRNA (XM_006355444). There are no transcripts spanning this region that have been identified in potato, while one EST has been identified comprising only the second exon region (JG563892). In either case the integration is not occurring in a coding region.

### Breeding outcomes and late blight resistance screening

Crosses proved successful when CBD 1 transgenic Bintje was the female parent. Transgenic Bintje remained male sterile due to the same lack of viable pollen as found in the controls. Crosses made to Bintje control flowers with the same fertile donor pollen, resulted in floral abscission, the usual phenotype. To test for successful breeding, a limited number of crosses were made to highlight the potential for new variety development.

**Fig. 5:**
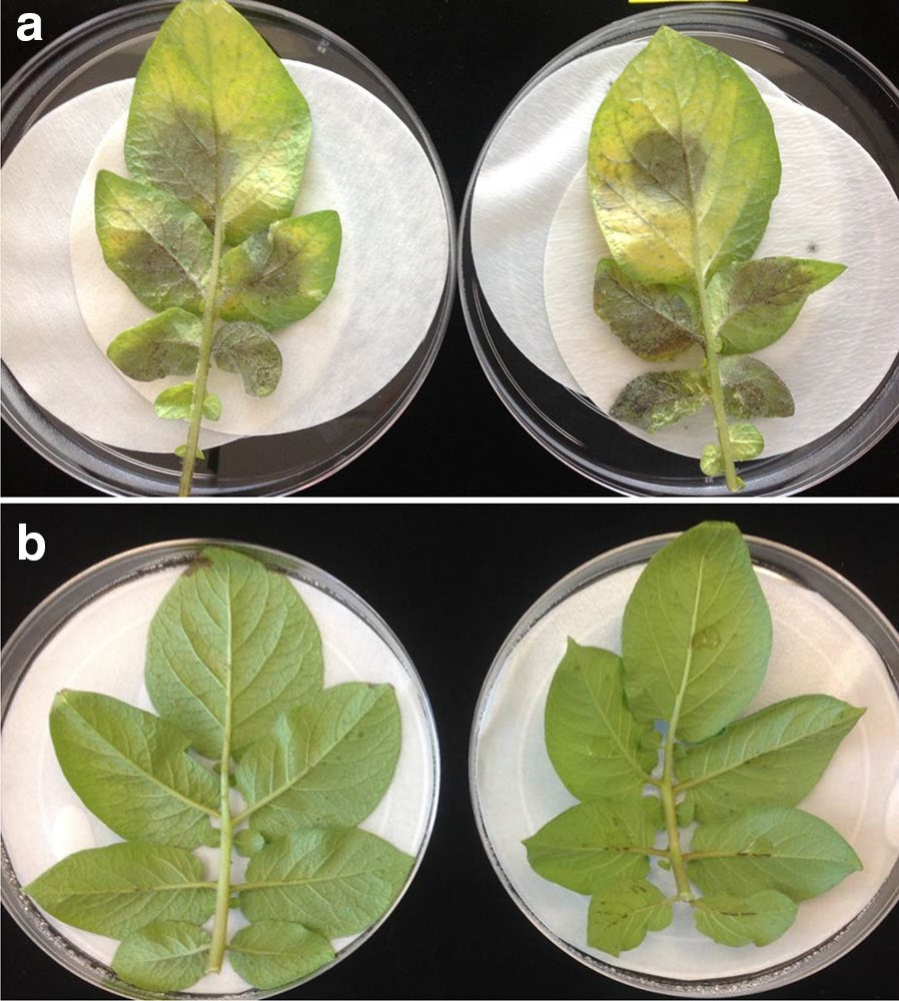
Detached leaflets were inoculated with *Phytophthora infestans* sporangia and zoospores, then incubated for 6 days. **a** Bintje controls are fully susceptible to *P. infestans* US 11. **b** Sarpo Mira is fully resistant. A subset of progeny from the B7x Sarpo Mira cross is fully resistant to late blight as shown in Table 1

**Fig. 6:**
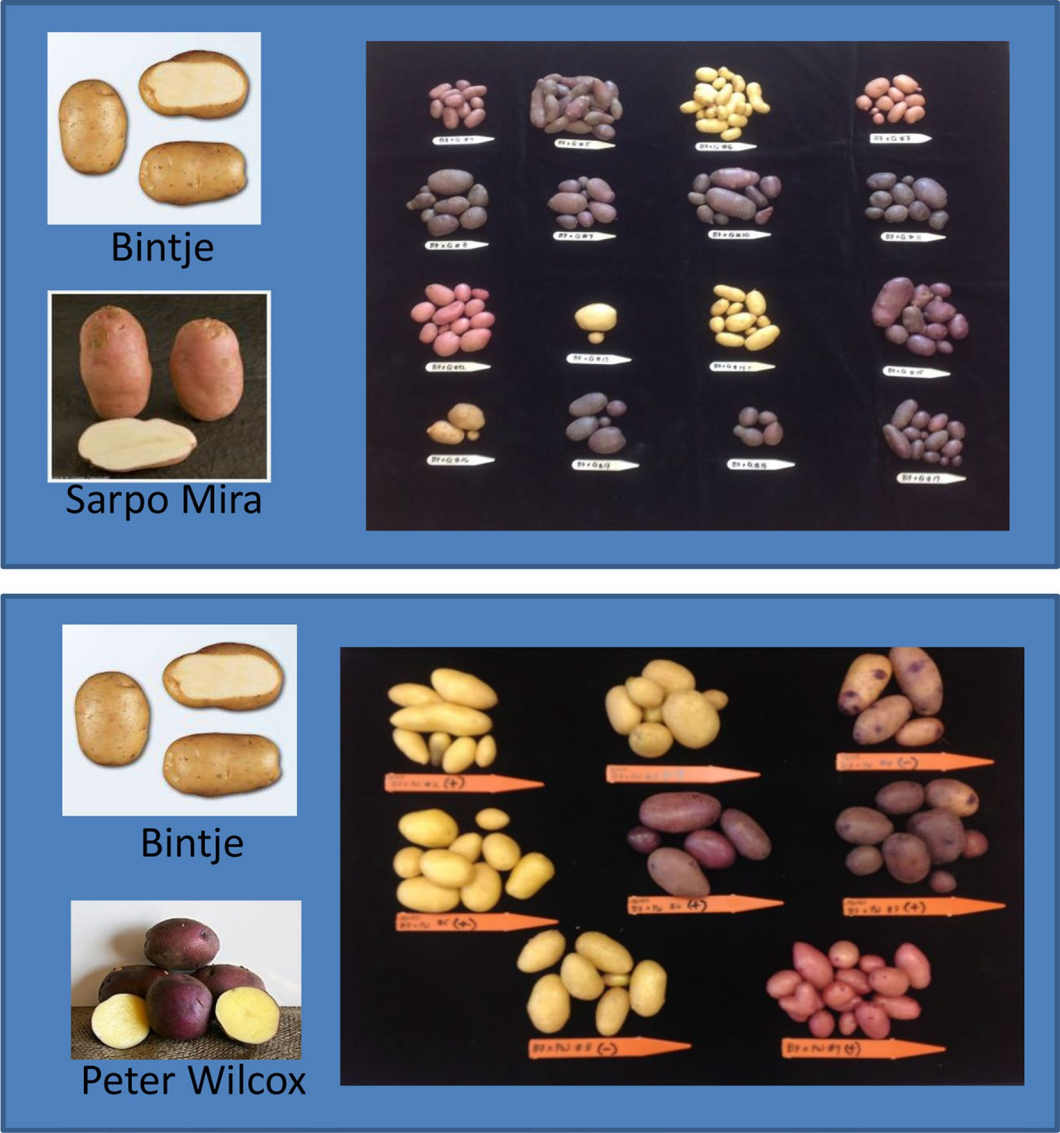
Cross between CBD 1 transgenic Bintje B7 and cultivar Sarpo Mira resulted in progeny with a wide range of tuber colors, suggesting complementation of an anthocyanin pathway. Cross between CBD 1 transgenic Bintje B7 and cultivar Peter Wilcox resulted in a range of *tuber shapes* and *colors*, representing potential for development of Bintje

There were no differences in the late blight susceptibility of any of the regenerated CBD 1 transgenic Bintjes, all were highly susceptible, like control Bintjes. Resistance to late blight was transferred into Bintje after crossing with Sarpo Mira (Fig. 5; Table 1). Complementation of the anthocyanin pathway is suggested in crosses between Bintje and Sarpo Mira, resulting in purple tubers in some progeny. Multigenic quality traits were successfully demonstrated in progeny after crossing pollen from the purple skin, yellow flesh Peter Wilcox cultivar to the CBD1 transgenic Bintje (Fig. 6).

**Table 1:** Disease screening for late blight resistance 6 days after inoculation with *Phytophthora infestans*

Potato line	Late blight rating
Bintje B7	Susceptible
Sarpo Mira	Resistant
B7SM 1	Resistant
B7SM 2	Resistant
B7SM 3	Mod Susc
B7SM 4	Resistant
B7SM 5	Resistant
B7SM 6	Resistant
B7SM 7	Resistant
B7SM 8	Resistant
B7SM 9	Resistant
B7SM 10	Resistant
B7SM 11	Mod Susc
B7SM 12	Resistant
B7SM 13	Resistant
B7SM 14	Resistant
B7SM 15	Mod Susc
B7SM 16	Resistant

## Discussion

Our original hypothesis was that introducing CBD 1 into transgenic potato might alter the host: pathogen interaction. One way this could happen would be if CBD 1 acted in a manner similar to *Cladosporium fulvum* AVR4, where the chitin binding AVR4 protein protects the hyphae from plant chitinases, preventing release of elicitor fragments [9, 10]. In the soybean: *P. sojae* interaction, soybean produces glucanases that attack the cellulosic *P. sojae* hyphae, releasing elicitor fragments. CBD 1 protein could be one mechanism for protecting the hyphae, along with the reported glucanase inhibitor proteins [11]. This would lead to greater host susceptibility, but this was not seen in our studies.

An alternative possibility would be that CBD 1 interacts with the host cell wall. Interactions with the host cell wall could increase susceptibility if the CBD acted to loosen cellulose microfibrils, or reduce susceptibility if the CBD bound to and shielded substrates susceptible to pathogen enzymes. While there was no evidence that these mechanisms were active, based on indistinguishable differences in susceptibility between control and transgenic CBD plants, there was an obvious effect on gross plant morphology.

There is evidence to suggest that CBDs can interact with the plant cell wall. This is seen in the use of binding modules as molecular probes of cell wall structure [3, 4]. It is also suggested in the limited reports of their use in transgenic plants. In one case, a family 3 CBD protein from the saprophytic bacterium *Clostridium cellulovorans* [12], was found to increase early growth of transgenic Desiree potato plants [13, 14]. At higher copy numbers there was an inhibition of growth. Interestingly, our use of a family 1 CBD resulted in greater growth only at later stages of plant development, and allowed for ovary development. The use of a Family 22 xylan binding module in transgenic tobacco failed to show any apparent effects on morphology or growth rates [15], while another study showed a marked reduction in growth of tobacco when a tandem CBM was expressed in transgenic materials [16]. Given the variable effects of CBDs in transgenic plants, there can be no assumptions made about the effect of any carbohydrate binding modules until tested.

The unusual feature of the Phytophthora CBD 1 expressing transgenic Bintje is the ovary formation. Even in the absence of fertilization the transgenic plants are able to produce berries. This would be similar to parthenocarpic fruit development, where fruit develops in the absence of seed development. This has been engineered into Solanaceous crops through manipulation of tissue-targeted auxin production [17].

Auxin has a well known role in causing cell wall loosening and cell expansion, through induction of various plant-encoded cell wall carbohydrate modifying enzymes [18]. After successful fertilization, the Bintje seed berries are larger than those produced without fertilization, indicating a normal interaction between seed development and ovary growth. The impact of CBD 1 on ovary development is unique as it has never been seen in other transgenic Bintjes, including the Phytophthora CBD4 (FJ524852) which encodes a double, or tandem, cellulose binding region (unpublished data). This discovery provides a new model for understanding ovary development in relation to cell wall proteins. It is of interest that Bintje can act as a female parent, once the block to ovary development is overcome. Further investigations may provide a genetic basis for this lack of ovary formation within the Bintje genome. Another use for this information may be in the development of tomato fruits. The developing fruit of tomato is the equivalent of the potato seed berry, thus there may be methods for producing fruits without pollination, or producing modified fruit shapes through use of CBD 1.

The mechanism of CBD-mediated ovary development remains to be characterized, however, CBD expression can be a useful tool for developing the Bintje gene pool, one that hasn’t changed since 1904 [19]. The cultivar Bintje is one of the most widely grown European cultivars (http://www.europotato.org) due to strong yields, growth under variable conditions, and excellent flavor under different cooking methods. Cultivar weakness is seen in a lack of resistance to many major pathogens of potato. We demonstrate the ability to integrate multigenic late blight resistance, as well as nutrition-relevant color changes, into the Bintje gene pool. Selection can be made for progeny of transgenic Bintje that lack the *CBD* 1 transgene. The primary value of CBD 1 expression is in breeding. The progeny segregating for loss of the *CBD* 1 revert to the sterile form of the original Bintje cultivar. This will allow for improved Bintje without carryover of transgenes, for those markets where that is desired. Initial experiments indicate that the *CBD* 1 gene can also be used in other potatoes lacking female fertility, such as Russet Burbank, although initial progeny have not been as vigorous (unpublished).

## Conclusions

Expression of cellulose binding domain protein resulted in development of ovaries in the sterile potato cultivar Bintje. Ovaries formed in the absence of fertilization, while larger ovaries were formed after outcrossing with fertile pollen and subsequent seed production. This opens the possibility for restoring ovary formation to other sterile germplasm, as well as the possibility of modifying the production of consumable ovaries such as tomatoes.
